# Synthesis and Anti-Cancer Activity of New Pyrazolinyl-Indole Derivatives: Pharmacophoric Interactions and Docking Studies for Identifying New EGFR Inhibitors

**DOI:** 10.3390/ijms23126548

**Published:** 2022-06-11

**Authors:** Habibullah Khalilullah, Deepak K. Agarwal, Mohamed J. Ahsan, Surender S. Jadav, Hamdoon A. Mohammed, Masood Alam Khan, Salman A. A. Mohammed, Riaz Khan

**Affiliations:** 1 Department of Pharmaceutical Chemistry and Pharmacognosy, Unaizah College of Pharmacy, Qassim University, Unaizah 51911, Saudi Arabia; 2 Department of Pharmaceutical Chemistry, Alwar College of Pharmacy, Alwar 302 023, Rajasthan, India; deepakkumar753@gmail.com; 3 Department of Pharmaceutical Chemistry, Maharishi Arvind College of Pharmacy, Jaipur 302 023, Rajasthan, India; jawedpharma@gmail.com; 4 Centre for Molecular Cancer Research (CMCR), Vishnu Institute of Pharmaceutical Education and Research (VIPER), Narsapur 502 313, Telangana, India; surenderjs@gmail.com; 5 Department of Medicinal Chemistry and Pharmacognosy, College of Pharmacy, Qassim University, Qassim 51452, Saudi Arabia; ham.mohammed@qu.edu.sa (H.A.M.); kahn.r.a@gmx.com (R.K.); 6 Department of Pharmacognosy and Medicinal Plants, Faculty of Pharmacy, Al-Azhar University, Cairo 11884, Egypt; 7 College of Applied Sciences, Qassim University, Qassim 51452, Saudi Arabia; masakhan@gmail.com; 8 Department of Pharmacology and Toxicology, College of Pharmacy, Qassim University, Qassim 51452, Saudi Arabia; salmanafroze1@gmail.com

**Keywords:** anti-cancer, tyrosine kinase, indole, pyrazole, pyrazolinyl-indole, EGFR, cell lines, molecular modeling, ligand docking

## Abstract

Newly designed series of indole-containing pyrazole analogs, pyrazolinylindoles, were synthesized, and their structures were confirmed based on the spectral data of the ^1^H NMR, ^13^C NMR, and HR-MS analyses. Preliminary anti-cancer activity testings were carried out by the National Cancer Institute, United States of America (NCI, USA). Compounds HD02, HD05, and HD12 demonstrated remarkable cytotoxic activities against nine categories of cancer types based cell line panels which included leukemia, colon, breast, melanoma, lungs, renal, prostate, CNS, and ovarian cancer cell lines. The highest cytotoxic effects were exhibited by the compounds HD02 [1-(5-(1-H-indol-3-yl)-3-(p-tolyl)-4,5-dihydro-1H-pyrazol-1-yl)-2-phenylethanone], HD05 [1-(3-(4-chlorophenyl)-5-(1H-indol-3-yl)-4,5-dihydro-1H-pyrazol-1-yl)-2-phenoxyethanone], and HD12 [(3-(4-chlorophenyl)-5-(1H-indol-3-yl)-4,5-dihydro-1H-pyrazol-1-yl)(pyridin-4-yl)methanone] against some of the 56 types of NCI-based cell lines in different panels. Compound HD05 showed the maximum range of cancer cell growth inhibitions against all categories of the cell lines in all nine panels. On average, in comparison to the referral standard, imatinib, at a dose level of 10 µM, the HD05 showed significant activity against leukemia in the range of 78.76%, as compared to the imatinib at 9% of cancer cells’ growth inhibitions. Molecular docking simulation studies were performed in silico on the epidermal growth factor receptor (EGFR) tyrosine kinase, in order to validate the activity.

## 1. Introduction

Cancer is a global concern and draws major attention from governments worldwide. The World Health Organization (WHO) also published interval statistical reports for cancer incidences, preventions, and management protocols [1]. According to the latest WHO estimates, the global cancer burden is set to rise by over 18 million new cases per year for the next decade, and to reach nearly 9.6 million deaths during the period [1]. Several factors, including environmental, lifestyle-, and physiology-related, are involved in an ever-increasing number of cancer cases [2]. Aging and the challenging prevalence of causes of cancers linked to dietary, nutritional, and other health-related conditions are also paramount to contributing to its incidences. Lung and breast cancers are the most common types in terms of the number of evolving types, and new cases are reported each year. Colorectal cancer is the third-most commonly diagnosed cancer in the United States [1,3]. A large number of anti-cancer drugs have developed resistance, and serious adverse effects of radiation and chemotherapy that exhibit alopecia and bone marrow depression are major drawbacks. Metastatic cancer recurrence necessitates the design and development of new cancer therapies through the development of newer structural templates and molecular leads [4]. Several enzymatic targets have been explored, and kinases have been observed to be playing important roles in various processes of cellular growth, proliferation, differentiation, motility, and cell survival. The proliferation is primarily attributed to kinases known as EGFR-TK (Epidermal Growth Factor Receptor-Tyrosine Kinase), which form part of the family of tyrosine kinases, and are currently considered an important target in the design and development of the new anti-cancer agents. Several molecular entities belonging to the O- and N-heterocyclic frameworks have been proposed, and among them, the pyrazolines are an imperative structural motif that possesses a wide range of biological activities against different cancer types [5,6,7,8,9,10], as well as other pharmacological activities [11,12,13,14,15,16,17,18,19]. The current approach aimed towards designing new templates spear-headed on the fragment-based drug design concept approach, wherein the targets were selected based on their fragmented molecular framework, and the summative effects of the ligand on the target protein were considered. The sub-structural fragments that exhibited different levels of anti-cancer properties and binding feasibility at the target site were chosen to proceed with. The current work is an attempt to explore the possible role of EGFR-TKs through the in silico modeling approach. Based on preliminary receptor docking studies, the receptor-ligand interactions feasibility and binding domain functions are proposed for a presumed elicitation of growth inhibitions of certain cancer cell lines. The concurrent study does not dwell upon the EGFR kinase inhibition assay, but solely provides the modeling and cell-line based approaches towards this end. Nonetheless, the EGFR is an important target in clinical oncology, and several EGFR inhibitors have been developed and clinically approved in recent years, which include small molecule, non-monoclonal antibody (non-mAb)-targeted, TK-acting drug candidates, e.g., imatinib, gefitinib, erlotinib, icotinib, and most recently osimertinib, among others [20].

## 2. Materials and Methods

The reactions were monitored by TLC (Thin Layer Chromatography) using an *n*-hexane and ethyl acetate mixture. Iodine vapors were used for visualization. All the melting points were determined in an open glass capillary using liquid paraffin, and are uncorrected. The purity of all the products were determined by TLC, and were used after crystallization. The IR spectra were recorded on a Bruker spectrometer. The mass spectra were recorded on a Bruker Daltronics high-resolution mass spectrometer. The ^1^H-NMR were recorded at 400 MHz using a Bruker 400 MHz (Avance) instrument with tetramethylsilane as the internal standard. Elemental analyses were performed on Elementar Vario EL III, Carlo Erba 1108. 

### 2.1. Chemical Synthesis

General procedure for the synthesis of acid hydrazides (**1a**–**c**): A mixture of aromatic acid (1 mmol) and ethanol (10 mmol, excess) in acidic medium was refluxed for 4–6 h. After completion of the ester formation, the mixture was cooled, and a solution of sodium bicarbonate was added to neutralize the excess acid in the reaction mixture. Hydrazine hydrate was added to the mixture, and the mixture was further refluxed for 17–22 h. After completion of the reaction, the mixture was concentrated to dryness, the solvent was removed, and the reaction mixture was cooled to solidify the product that was washed with water, air-dried, and kept for further use. 

### 2.2. General Procedure for the Synthesis of *1*-(*2*-Aryl)-*3*-(*1*-H-indole-*3*-yl)prop-*2*-en-*1*-on (***2a***–***d***)

An equimolar quantity of indole-3-carboxaldehyde and corresponding acetophenone derivative was mixed in ethanol (30 mL); then, 2 mL of 10% NaOH added, and the mixture was stirred for 4–6 hr. Progress of the reaction was monitored by TLC. After completion, the crude product was poured into crushed ice, and the precipitate was collected, washed with water, and dried. The chalcone product (2d) 1-(4-chloro-phenyl)-3-(1H-indole-3-yl)-prop-2-en-1-one obtained as yellow solid (2.56 g, 70.00%) was analyzed, m.p. 146–149 °C; IR absorbance (cm^−1^) 2947–3093.6 (C–H, str, aromatic), 1663 (C=O, str), 1542 (C=C, str),1284 (C–N, str), 675 (C–Cl, str); ^1^H-NMR (400 MHz, DMSO-d_6_) δ_H_: 9.99 (1H, s, indole –NH), 8.22 (2H, d, *J* = 6.0 Hz, Ar–H Ring B), 7.62 (2H, d, *J* = 9.0 Hz, Ar–H Ring B), 7.29–7.43 (4H, m, Ar–H ring A). 

### 2.3. General Procedure for the Synthesis of (Substituted Aryl)-Pyrazolines (HD01–HD12)

An equimolar mixture of the corresponding chalcone and acid hydrazide derivative in glacial acetic acid (25 mL) was refluxed for 18–22 hr. After completion of the reaction as evident from the TLC, the mixture was concentrated to dryness, cooled at room temperature, and poured into 50 mL of (distilled) water. The obtained solid precipitate was filtered, washed with water, and air-dried to obtain the title compounds which were purified by crystallization using acetone-ethanol. 

#### 2.3.1. 1-(3-(4-Hydroxyphenyl)-5-(1*H*-indol-3-yl)-4,5-dihydro-1*H*-pyrazol-1-yl)-2-phenylethanone, [HD01]

Dark brown solid (0.48 g, 67.80%), m.p. 146–48 °C; IR absorptions (cm^−1^); 3749 (N-H, str), 3174.6–2977.9 (C–H, str, aromatic), 1655 (C=O, str), 1558 (C=N, str), 1265 (C–N, str), 1161 (C–O, str), 745.5 (aromatic mono-substituted); ^1^H-NMR (400 MHz, DMSO-d_6_): δ_H_ 9.92 (1H, s, indole –NH), 8.29 (2H, d, J = 5.5 Hz, Ar–H), 8.08 (2H, d, *J* = 5.95 Hz, Ar–H), 7.20–7.92 (7H, m, Ar–H), 5.45 (1H, dd, *J*_AB_ = 17.5 Hz, *J*_AC_ = 4.3 Hz, *J*_BC_ = 10.8 Hz, CH_C_ of pyrazoline), 3.54 (1H, dd, *J*_AB_ = 17.5 Hz, *J*_BC_ = 10.8 Hz, CH_2_-H_B_ of pyrazoline), 3.25 (1H, dd, *J*_AB_ = 17.5 Hz, *J*_AC_ = 2.8 Hz, CH_2_-H_A_ of pyrazoline), 3.12 (2H, s, COCH_2_); ^13^C–NMR (100 MHz, DMSO-d_6_) δ (ppm): 164.85 (C, C=O), 151.67 (C, pyrazoline C3), 149.45 (C, aromatic), 146.23 (CH, aromatic), 140.95 (C, aromatic), 134.34 (C, aromatic), 132.59 (CH, aromatic), 131.45 (CH, aromatic), 130.58 (C, aromatic), 129.24 (2CH, aromatic), 128.56 (CH, aromatic), 128.32 (2CH, aromatic), 60.124 (CH, pyrazoline C_5_), 43.35 (CH_2_, pyrazoline C_4_); HRMS (*m*/*z*): 396.1605 [M + H]^+.^ Anal. Calcd. for C_22_H_17_N_3_O_2_S: C, 68.20 H, 4.42, N, 10.85. Found: C, 68.15; H, 4.48, N, 10.82.

#### 2.3.2. 1-(5-(1-H-Indol-3-yl)-3-(p-tolyl)-4,5-dihydro-1*H*-pyrazol-1-yl)-2-phenylethanone, [HD02]

Yellowish brown solid (0.46 g, 60.50%), m.p. 145–147 °C; IR absorptions (cm^−1^); 3750 (N-H, str), 3175–2989 (C–H, str, aromatic), 1660 (C=O, str), 1565 (C=N, str), 1272 (C–N, str), 1165 (C–O, str), 748 (aromatic monosub); ^1^H-NMR (400 MHz, DMSO-d_6_): δ_H_ 9.84 (1H, s, Indole –NH), 8.19 (2H, d, *J* = 5.5 Hz, Ar–H), 8.10 (2H, d, *J* = 5.9 Hz, Ar–H), 7.21–7.95 (8H, m, Ar–H), 5.17 (1H, dd, *J_AB_* = 18.7 Hz, *J_AC_* = 2.4 Hz, *J_BC_* = 10.9 Hz, CH– of pyrazoline), 3.65 (1H, dd, *J_AB_* = 18.7 Hz, *J_BC_* = 10.9 Hz, CH_2_-H_B_ of pyrazoline), 3.49 (1H, dd, *J_AB_* = 18.7 Hz, *J_AC_* = 2.4 Hz, CH_2_-H_A_ of pyrazoline), 3.15 (2H, s, COCH_2_), 2.54 (3H, s, Ar-CH_3_); ^13^C–NMR (100 MHz, DMSO-d_6_) δ (ppm): 152.85 (C, C=O), 151.56 (C, pyrazoline C3), 148.23 (C, aromatic), 145.26 (CH, aromatic), 140.85 (C, aromatic), 134.65 (C, aromatic), 132.34 (CH, aromatic), 131.15 (CH, aromatic), 130.35 (C, aromatic), 129.15 (2CH, aromatic), 128.35 (CH, aromatic), 127.32 (2CH, aromatic), 62.24 (CH, pyrazoline C_5_), 44.25 (CH_2_, pyrazoline C_4_); HRMS (*m*/*z*): 393.1838 [M + H]^+^: Anal. Calcd for C_26_H_23_N_3_O (393.1841): C, 79.36; H, 5.89; N, 10.68; O, 4.07 Found: C, 75.46; H, 5.92, N, 9.82.

#### 2.3.3. 1-(3-(2-Hydroxyphenyl)-5-(1*H*-indol-3-yl)-4,5-dihydro-1*H*-pyrazol-1-yl)-2-phenylethanone, [HD03]

Brown solid (0.38 g, 69.75%), m.p. 184–186 °C; IR absorptions (cm^−1^); 3705 (N-H, str), 3410 (O-H, str), 3152 (C–H, str, aromatic), 1792 (C=O, str), 1592 (C=N, str), 1288 (C–N, str), 1170 (C–O, str) 795 (aromatic monosub), 1498 (C=C, str), ^1^H-NMR (400 MHz, DMSO-d_6_) δ_H_: 9.75 (1H, s, Indole –NH), 9.15 (1H, s, Ar-OH), 8.25 (2H, d, J = 5.9 Hz, Ar–H), 8.19 (2H, d, *J* = 5.7 Hz, Ar–H), 7.07–7.92 (9H, m, Ar–H), 5.39 (1H, dd, J_AB_ = 17.1 Hz, *J*_AC_ = 2.5 Hz, J_BC_ = 11.1 Hz, CH-C of pyrazoline), 3.48 (1H, dd, *J*_AB_ = 17.6 Hz, *J*_BC_ = 11.5 Hz, CH_2_-H_B_ of pyrazoline), 3.41(1H, dd, *J*_AB_ = 17.6 Hz, *J*_AC_ = 2.5 Hz, CH_2_-_HA_ of pyrazoline), 2.85 (2H, s, CH_2_); ^13^C–NMR (100 MHz, DMSO-d_6_) δ (ppm): 152.65 (C, C=O), 152.46 (C, pyrazoline C3), 148.35 (C, aromatic), 144.56 (CH, aromatic), 142.25 (C, aromatic), 134.55 (C, aromatic), 132.45 (CH, aromatic), 132.15 (CH, aromatic), 130.55 (C, aromatic), 130.15 (2CH, aromatic), 126.42 (CH, aromatic),125.32 (2CH, aromatic), 60.24 (CH, pyrazoline C_5_), 45.15 (CH_2_, pyrazoline C_4_); HRMS (*m*/*z*): 395.1352 [M + H]^+^: Anal. Calcd for C_25_H_21_N_3_O (395.1634): C, 75.93; H, 5.35; N, 10.63; O, 8.09, Found: C, 75.56; H, 5.15, N, 10.82.

#### 2.3.4. 1-(3-(4-Chlorophenyl)-5-(1*H*-indol-3-yl)-4,5-dihydro-1*H*-pyrazol-1-yl)-2-phenylethanone, [HD04]

Brown solid (0.42 g, 78.10%), m.p. 158–160 °C; IR absorptions (cm^−1^); 3756 (N-H, str), 3164–2977 (C–H, str, aromatic), 1652 (C=O, str), 1530 (C=N, str), 1290 (C–N, str), 795 (aromatic monosub), 725 (C–Cl, str), ^1^H-NMR (400 MHz, DMSO-d_6_) δ_H_: 9.89 (1H, s, Indole –NH), 8.3 (2H, d, *J* = 5.8 Hz, Ar–H), 8.11(2H, d, *J* = 5.4 Hz, Ar–H), 6.98–7.98 (9H, m, Ar–H), 5.39 (1H, dd, *J*_AB_ = 17.0 Hz, *J*_AC_ = 2.7 Hz, *J*_BC =_ 11.4 Hz, CH_C_- of pyrazoline), 3.52 (1H, dd, *J*_AB_ = 17.0 Hz, *J*_BC_ = 11.4 Hz, CH_2_-H_B_ of pyrazoline), 3.42 (1H, dd, *J*_AB_ = 17.0 Hz, *J*_AC_ = 2.7 Hz, CH_2_-H_A_ of pyrazoline), 2.95 (2H, s, CH_2_); ^13^C–NMR (100 MHz, DMSO-d_6_) δ (ppm): 153.45 (C, C=O), 150.50 (C, pyrazoline C3), 148.65 (C, aromatic), 144.56 (CH, aromatic), 144.25 (C, aromatic),139.60 (C, aromatic),138.45 (CH, aromatic),138.15 (CH, aromatic), 36.55 (C, aromatic), 132.25 (2 CH, aromatic), 127.15 (CH, aromatic), 125.25 (2CH, aromatic), 59.25 (CH, pyrazoline C_5_), 46.25 (CH_2_, pyrazoline C_4_). HRMS (*m*/*z*): 413.8567 [M + H]^+^: Anal. Calcd for C_25_H_20_ClN_3_O (413.90) C, 72.55; H, 4.87; Cl, 8.57; N, 10.15; O, 3.87 Found C, 72.58; H, 4.83; N, 10.20.

#### 2.3.5. 1-(3-(4-Chlorophenyl)-5-(1*H*-indol-3-yl)-4,5-dihydro-1*H*-pyrazol-1-yl)-2-phenoxyethanone, [HD05]

Yellowish brown solid (0.42 g, 75.50%), m.p. 151–153 °C; IR absorptions (cm^−1^); 3754 (N-H, str), 3580 (O-H str), 2784 (C–H, str, aromatic), 1625 (C=O str), 1545 (C=N str), 1285 (C–N, str), 1180 (C–O, str), 810 (aromatic monosub), ^1^H-NMR (400 MHz, DMSO-d_6_): δ_H_: 9.95 (1H, s, Indole –NH), 8.27 (2H, d, *J* = 6.1 Hz, Ar–H), 7.62 (2H, d, *J* = 6.0 Hz, Ar–H), 8.11(2H, d, *J* = 6.00 Hz, Ar–H), 7.07–7.56 (7H, m, Ar–H), 5.39 (1H, dd, *J*_AB_ = 18.2 Hz, *J*_AC_ = 2.6 Hz, *J*_BC_ = 12.5 Hz, CH_C_- of pyrazoline), 4.9 (2H, s, –OCH_2_), 3.45 (1H, dd, *J*_AB_ = 18.2 Hz, *J*_BC_ = 12.5 Hz, CH_2_-H_B_ of pyrazoline), 3.36 (1H, dd, *J*_AB_ = 18.2 Hz, *J*_AC_ = 2.6 Hz, CH_2_-H_A_ of pyrazoline), 3.15 (2H, s, CH_2_); ^13^C–NMR (100 MHz, DMSO-d_6_) δ (ppm): 152.56 (C, C=O), 150.50 (C, pyrazoline C3), 148.65 (C, aromatic), 144.56 (CH, aromatic), 144.25 (C, aromatic), 139.85 (C, aromatic), 138.62 (CH, aromatic), 138.25 (CH, aromatic), 136.25 (C, aromatic), 132.38 (2CH, aromatic), 125.35 (CH, aromatic), 123.55 (2CH, aromatic), 60.15 (CH, pyrazoline C_5_), 46.45 (CH_2_, pyrazoline C_4_); HRMS (*m*/*z*): 429.1235 [M + H]^+^: Anal. Calcd for C_25_H_20_ClN_3_O_2_ (429.14) C, 69.85; H, 4.69; Cl, 8.25; N, 9.77; Found C, 70.15, H, 4.87; N, 10.15.

#### 2.3.6. 1-(5-(1-H-Indol-3-yl)-3-(p-tolyl)-4,5-dihydro-1*H*-pyrazol-1-yl)-2-phenoxyethanone, [HD06]

Brownish solid (0.42 g, 74.80%), m.p. 154–157 °C; IR absorptions (cm^−1^); 3695 (N-H, str), 3174–2977 (C–H, str, aromatic), 3050 (C–H str. aliphatic), 1656 (C=O str), 1556 (C=N str), 1290 (C–N str), 1135 (C–O str), 795 (Aromatic monosub). ^1^H-NMR (400 MHz, DMSO-d_6_) δ_H_: 9.75 (1H, s, Indole –NH), 8.31 (2H, d, *J* = 5.75 Hz, Ar–H), 8.11(2H, d, *J* = 5.9 Hz, Ar–H), 7.55 (2H, d, *J* = 5.6 Hz, Ar–H), 6.56–7.25 (8H, m, Ar–H), 5.24 (1H, dd, *J*_AB_ = 16.5 Hz, *J*_AC_ = 2.8 Hz, *J*_BC =_ 12.5 Hz, CH_C_- of pyrazoline), 3.62 (1H, dd, *J*_AB_ = 16.5 Hz, *J*_BC_ = 12.5 Hz, CH_2_-H_B_ of pyrazoline),3.45 (1H, dd, *J*_AB_ = 16.5 Hz, *J*_AC_ = 2.8 Hz, CH_2_-H_A_ of pyrazoline), 3.10 (2H, s, CH_2_), 2.35 (3H, s, Ar-CH_3_); ^13^C–NMR (100 MHz, DMSO-d_6_) δ (ppm): 153.64 (C, C=O), 150.35 (C, pyrazoline C3), 147.85 (C, aromatic), 145.25 (CH, aromatic), 144.80 (C, aromatic), 140.55 (C, aromatic), 139.25 (CH, aromatic), 138.36 (CH, aromatic), 137.40 (C, aromatic), 133.35 (2CH, aromatic), 125.45 (CH, aromatic), 120.65 (2CH, aromatic), 59.25 (CH, pyrazoline C_5_), 47.25 (CH_2_, pyrazoline C_4_); HRMS (*m*/*z*): 409.1456 [M + H]^+^: Anal. Calcd for C_26_H_23_N_3_O_2_ (409.47) C, 76.26; H, 5.66; N, 10.26; O, 7.81, Found C, 76.15, H, 5.25; N, 9.85.

#### 2.3.7. 1-(3-(2-Hydroxyphenyl)-5-(1*H*-indol-3-yl)-4,5-dihydro-1*H*-pyrazol-1-yl)-2-phenoxyethanone, [HD07]

Yellowish solid (51.20%), m.p. 171–174 °C; IR absorptions (cm^−1^); 3650 (N-H, str), 3388 (O-H, str), 2975.9 (C–H, str, aromatic), 1652 (C=O, str), 1562 (C=N, str), 1292 (C–N, str), 1170 (C–O, str), 795 (aromatic monosub), ^1^H-NMR (400 MHz, DMSO-d_6_) δ_H_: 9.8 (1H, br s, NH; exchangeable with D_2_O), 9.25 (1H, s, Ar-OH), 8.25 (2H, d, *J* = 6.5 Hz, Ar–H), 8.11 (2H, d, *J* = 6.5 Hz, Ar–H), 6.85–7.83 (7H, m, Ar–H), 5.26 (1H, dd, *J*_AB_ = 17.8 Hz, *J*_AC_ = 2.8 Hz, *J*_BC =_ 11.3 Hz, CH_C_- of pyrazoline), 4.40 (2H, s, OCH_2_), 3.55 (1H, dd, *J*_AB_ = 17.8 Hz, *J*_BC_ = 11.3 Hz, CH_2_-H_B_ of pyrazoline), 3.45 (1H, dd, *J*_AB_ = 17.8 Hz, *J*_AC_ = 2.8 Hz, CH_2_-H_A_ of pyrazoline); ^13^C–NMR (100 MHz, DMSO-d_6_) δ (ppm): 145.25 (CH, aromatic), 142.65 (C, aromatic), 140.85 (C, aromatic), 139.55 (CH, aromatic), 138.45 (CH, aromatic), 136.75 (C, aromatic), 132.25 (2CH, aromatic), 124.55 (CH, aromatic), 121.45 (2CH, aromatic), 57.25 (CH, pyrazoline C_5_), 48.25 (CH_2_, pyrazoline C_4_); HRMS (*m*/*z*): 411.1452 [M + H]^+^: Anal. Calcd for C_26_H_23_N_3_O_2_ (411.45) C, 72.98; H, 5.66; N, 10.21, Found C, 73.45, H, 5.45; N, 9.65.

#### 2.3.8. Phenyl-3-(4-hydroxyphenyl)-5-(1*H*-indol-3-yl)-4,5-dihydro-1*H*-pyrazole-1-carboxylate, [HD08]

Yellow solid (0.42 g, 74.0%), m.p. 165–168 °C; IR absorptions (cm^−1^); 3750 (N-H, str), 3365 (O-H, str), 3165.6–2985 (C–H, str, aromatic), 1635 (C=O str), 1530 (C=N, str), 1282 (C–N, str), 1165 (C–O, str) 799 (aromatic monosub), ^1^H-NMR (400 MHz, DMSO-d_6_) δ_H_: 9.85 (1H, br s, NH; exchangeable with D_2_O), 8.95 (1H, s, Ar-OH), 8.31(2H, d, *J* = 5.8 Hz, Ar–H), 8.11 (2H, d, *J* = 5.5 Hz, Ar–H), 3.25 (1H, dd, *J*_AB_ = 17.9 Hz, *J*_AC_ = 3.5 Hz, CH_2_-H_A_ of Pyrazoline), 7.15–7.52 (8H, m, Ar–H), 4.26 (s, 2H, OCH_2_) 5.25 (1H, dd, *J*_AB_ = 17.9 Hz, *J*_AC_ = 3.5 Hz, *J*_BC =_ 11.2 Hz, CH_C_ of Pyrazoline), 3.45 (1H, dd, *J*_AB_ = 17.9 Hz, *J*_BC_ = 11.2 Hz, CH_2_-H_B_ of Pyrazoline); ^13^C–NMR (100 MHz, DMSO-d_6_) δ (ppm): 143.75 (C, aromatic), 142.50 (CH, aromatic), 140.55 (C, aromatic), 138.75 (CH, aromatic), 136.25 (CH, aromatic), 135.55 (C, aromatic), 130.25 (2CH, aromatic), 122.25 (CH, aromatic), 118.25 (2CH, aromatic), 56.5 (CH, pyrazoline C_5_), 48.35 (CH_2_, pyrazoline C_4_), HRMS (*m*/*z*): 411.1625 [M + H]^+^: Anal. Calcd for C_25_H_21_N_3_O_3_ (411.45) C, 72.98; H, 5.14; N, 10.21; Found C, 73.25, H, 5.23; N, 9.85.

#### 2.3.9. (3-(4-Hydroxyphenyl)-5-(1*H*-indol-3-yl)-4,5-dihydro-1*H*-pyrazol-1-yl)(pyridin-4-yl)methanone, [HD09]

Yellow solid (0.42 g, 57.50%), m.p. 168–171 °C; IR absorptions (cm^−1^); 3750 (N-H, str), 3362 (O-H, str), 3172 (C–H, str, aromatic), 1636 (C=O, str), 1530 (C=N, str), 1285 (C–N, str), 1165 (C–O, str) 802 (aromatic), ^1^H-NMR (400 MHz, DMSO-d_6_) δ_H_: 9.54 ((1H, br s, NH; exchangeable with D_2_O), 8.90 (1H, s, Ar-OH); 8.31(2H, d, *J* = 5.8 Hz, Ar–H), 7.10–7.62 (8H, m, Ar–H), 5.12 (1H, dd, *J*_AB_ = 17.95 Hz, *J*_AC_ = 3.2 Hz, *J*_BC =_ 11.2 Hz, CH_C_- of pyrazoline), 3.54 (1H, dd, *J*_AB_ = 17.5 Hz, *J*_BC_ = 11.2 Hz, CH_2_-H_B_ of pyrazoline), 3.45 (1H, dd, *J*_AB_ = 17.5 Hz, *J*_AC_ = 3.2 Hz, CH_2_-H_A_ of pyrazoline); ^13^C–NMR (100 MHz, DMSO-d_6_) δ (ppm): 148.50 (CH, aromatic), 145.25 (C, aromatic), 142.45 (C, aromatic), 140.6 (CH, aromatic), 136.2 (CH, aromatic), 132.5 (C, aromatic), 129.5 (2CH, aromatic), 118.4 (CH, aromatic), 110.5 (2CH, aromatic), 56.3 (CH, pyrazoline C_5_), 45.5 (CH_2_, pyrazoline C_4_); HRMS (*m*/*z*): 383.1305 [M + H]^+^: Anal. Calcd for C_23_H_18_N_4_O_2_ (382.41) C, 72.24; H, 4.74; N, 14.65; O (8.37), Found C, 72.56, H, 5.15; N, 13.56.

#### 2.3.10. (5-(1-H-Indol-3-yl)-3-(p-tolyl)-4,5-dihydro-1*H*-pyrazol-1-yl)(pyridin-4-yl)methanone, [HD10]

Yellow solid (0.42 g, 52.33%), m.p. 161–163 °C; IR absorptions (cm^−1^); 3760.5 (N-H, str), 2982.5 (C–H str, aromatic), 1645.5 (C=O, str), 1548.2 (C=N, str), 1256.6 (C–N, str), 810.5 (aromatic), ^1^H-NMR (400 MHz, DMSO-d_6_) δ_H_: 9.25 (1H, br s, NH; exchangeable with D_2_O), 8.25 (2H, d, *J* = 6.5 Hz, Ar–H), 8.10 (2H, d, *J* = 6.5 Hz, Ar–H), 5.10 (1H, dd, *J*_AB_ = 17.5 Hz, *J*_AC_ = 2. 8 Hz, *J*_BC_ = 11.8 Hz, CH_C_- of pyrazoline), 7.2–7.5 (8H, m, Ar–H), 3.32 (1H, dd, *J*_AB_ = 17.5 Hz, *J*_BC_ = 10.5 Hz, CH_2_-H_B_ of pyrazoline), 3.25 (1H, dd, *J*_AB_ = 17.5 Hz, *J*_AC_ = 2.8 Hz, CH_2_-H_A_ of pyrazoline), 1.98 (3H, s, Ar-CH_3_); ^13^C–NMR (100 MHz, DMSO-d_6_) δ (ppm):, 145.5 (CH, aromatic), 142.5 (C, aromatic), 140.5 (C, aromatic), 138.5 (CH, aromatic), 136.5 (CH, aromatic), 132.5 (C, aromatic), 128.5 (2CH, aromatic), 112.5 (CH, aromatic), 110.5 (2CH, aromatic), 48.5 (CH, pyrazoline C_5_), 42.5 (CH_2_, pyrazoline C_4_); HRMS (*m*/*z*): 380.2305 [M + H]^+^: Anal. Calcd for C_24_H_20_N_4_O (380.44) C, 75.77; H, 5.30; N, 14.73; O (4.21), Found C, 74.65, H, 5.45; N, 13.85.

#### 2.3.11. (3-(2-Hydroxyphenyl)-5-(1*H*-indol-3-yl)-4,5-dihydro-1*H*-pyrazol-1-yl)(pyridin-4-yl)methanone, [HD11]

Yellowish brown solid (0.42 g, 69.66%), m.p. 175–178 °C; IR absorptions (cm^−1^); 3745.5 (N-H, str), 3450.5 (O-H, str), 3174.5–2953.9 (C–H str, aromatic), 1672.5 (C=O, str), 1552.5 (C=N, str), 1260.6 (C–N, str), 1165 (C–O, str) 807.5 (aromatic), ^1^H-NMR (400 MHz, DMSO-d_6_) δ_H_: 9.65 (1H, br s, NH; exchangeable with D_2_O), 8.91 (1H, s, Ar-OH, exchangeable with D_2_O); 8.26 (2H, d, *J* = 6.5 Hz, Ar–H); 8.10 (2H, d, *J* = 6.5 Hz, Ar–H); 7.12–7.35 (8H, m, Ar–H); 5.10 (1H, dd, *J*_AB_ = 16.8 Hz, *J*_AC_ = 2.5 Hz, *J*_BC_ = 11.50 Hz, CH_C_ of pyrazoline); 3.32 (1H, dd, *J*_AB_ = 16.8 Hz, *J*_BC_ = 11.5 Hz, CH_2_-H_B_ of pyrazoline), 3.25 (1H, dd, *J*_AB_ = 16.8 Hz, *J*_AC_ = 2.5 Hz, CH_2_-H_A_ of pyrazoline); ^13^C–NMR (100 MHz, DMSO-d_6_) δ (ppm): 143.5 (CH, aromatic), 142.5 (C, aromatic), 140.2 (C, aromatic), 136.4 (CH, aromatic), 134.5 (CH, aromatic), 130.2 (C, aromatic), 126.5 (2CH, aromatic), 111.3 (CH, aromatic), 108.6 (2CH, aromatic), 46.2 (CH, pyrazoline C_5_); 43.5 (CH_2_, pyrazoline C_4_); HRMS (*m*/*z*): 383.1245 [M + H]^+^: Anal. Calcd. for C_23_H_18_N_4_O_2_ (382.41) C, 72.24; H, 4.74; N, 14.65; O (8.37), Found C, 72.83, H, 5.23; N, 14.20.

#### 2.3.12. (3-(4-Chlorophenyl)-5-(1*H*-indol-3-yl)-4,5-dihydro-1*H*-pyrazol-1-yl)(pyridin-4-yl)methanone, [HD12]

Yellowish brown solid (0.42 g, 61.33%), m.p. 179–181 °C; IR absorptions (cm^−1^); 3730.5 (N-H, str), 3345.55 (O-H str), 3154.6–3005.9 (C–H str, aromatic), 1665.7 (C=O str), 1520.5 (C=N, str), 1240.5 (C–N, str), 1120 (C–O, str), 723.5 (aromatic), ^1^H-NMR (400 MHz, DMSO-d_6_) δ_H_: 9.2 (1H, br s, NH; exchangeable with D_2_O), 8.12 (2H, d, *J* = 7.5 Hz, Ar–H), 7.65 (2H, d, *J* = 7.5 Hz, Ar–H), 7.12–7.32 (8H, m, Ar–H), 5.15 (1H, dd, *J*_AB_ = 16.8 Hz, *J*_AC_ = 3.5 Hz, *J*_BC_ = 11.15 Hz, CH_C_- of pyrazoline), 3.25 (1H, dd, *J*_AB_ = 16.8 Hz, *J*_BC_ = 11.15 Hz, CH_2_-H_B_ of pyrazoline), 3.10 (1H, dd, *J*_AB_ = 16.8 Hz, *J*_AC_ = 3.2 Hz, CH_2_-H_A_ of pyrazoline); ^13^C–NMR (100 MHz, DMSO-d_6_) δ (ppm): 146.4 (CH, aromatic); 143.5 (C, aromatic), 142.2 (C, aromatic), 138.5 (CH, aromatic), 135.5 (CH, aromatic), 130.2 (C, aromatic),123.6 (2CH, aromatic), 112.5 (CH, aromatic), 103.5 (2CH, aromatic), 45.6 (CH, pyrazoline C_5_), 43.2 (CH_2_, pyrazoline C_4_), HRMS (*m*/*z*): 401.1125 [M + H]^+^: Anal. Calcd for C_23_H_17_ClN_4_O (400.86.41) C, 68.91; H, 4.27; N, 13.98; O (3.99), Found C, 69.53, H, 4.25; N, 14.15.

### 2.4. Anti-Cancer Screening Methodology

The initial in vitro cytotoxicities of the compounds were evaluated according to the one-dose protocol assay method against a panel of fifty-six tumor cell lines at the NCI (National Cancer Institute), Bethesda, USA. The origins and processing of the cell lines used in the study have been previously discussed [21,22,23,24]. Briefly, the human tumor cell line of the cancer screening panel was grown in RPMI 1640 medium containing 5% FBS (fetal bovine serum), and 2 mM L-glutamine. For a typical screening experiment, cells were inoculated into a 96-well microtiter plate in 100 μL at plating densities ranging from 5000 to 40,000 cells/well, depending upon the doubling time of the individual cell line. After cell inoculation, the microtiter plates were incubated at 37 °C, with 5% CO_2_, 95% air, and 100% relative humidity for 24 hr prior to the addition of the test compound. After 24 h, two plates of each cell line were fixated in situ with TCA (trichloroacetic acid), in order to represent the measurements of the cell populations for each cell line at the time of each test compound’s addition. The test compound was solubilized in DMSO (dimethyl sulfoxide) at 400x-folds of the desired final maximum test concentration, and was stored frozen prior to its use. At the time of test compound addition, an aliquot of frozen concentrate was thawed and was diluted to double the desired final maximum test concentration with complete medium, containing 50 μg/mL gentamicin. Following the test compound’s addition, the plate was incubated for an additional 48 hr at 37 °C, 5% CO_2_, 95% air, and 100% relative humidity. For the adherent cells, the assay was terminated by addition of cold TCA. The cells were fixated in situ by gentle addition of 50 μL of cold 50% (*w*/*v*) TCA (final concentration, 10% TCA), and incubated for 60 min at 4 °C. The supernatant was discarded, and the plate was washed five times with water and air-dried. The sulforhodamine B (SRB) solution (100 μL) at 0.4% (*w*/*v*) in 1% acetic acid was added to each well, and the plate was incubated for 10 min at room temperature. After staining, unbound dye was removed by washing five times with 1% acetic acid, and the plate was air-dried. Bound stain was subsequently solubilized with 10 mM trizma base, and the absorbance was read on an automated plate reader at a wavelength of 515 nm. For suspension cells, the methodology was the same except that the assay was terminated by fixing settled cells at the bottom of the wells by gently adding 50 μL of 80% TCA (final concentration, 16% TCA).

### 2.5. Molecular Modelling

**Figure 1:**
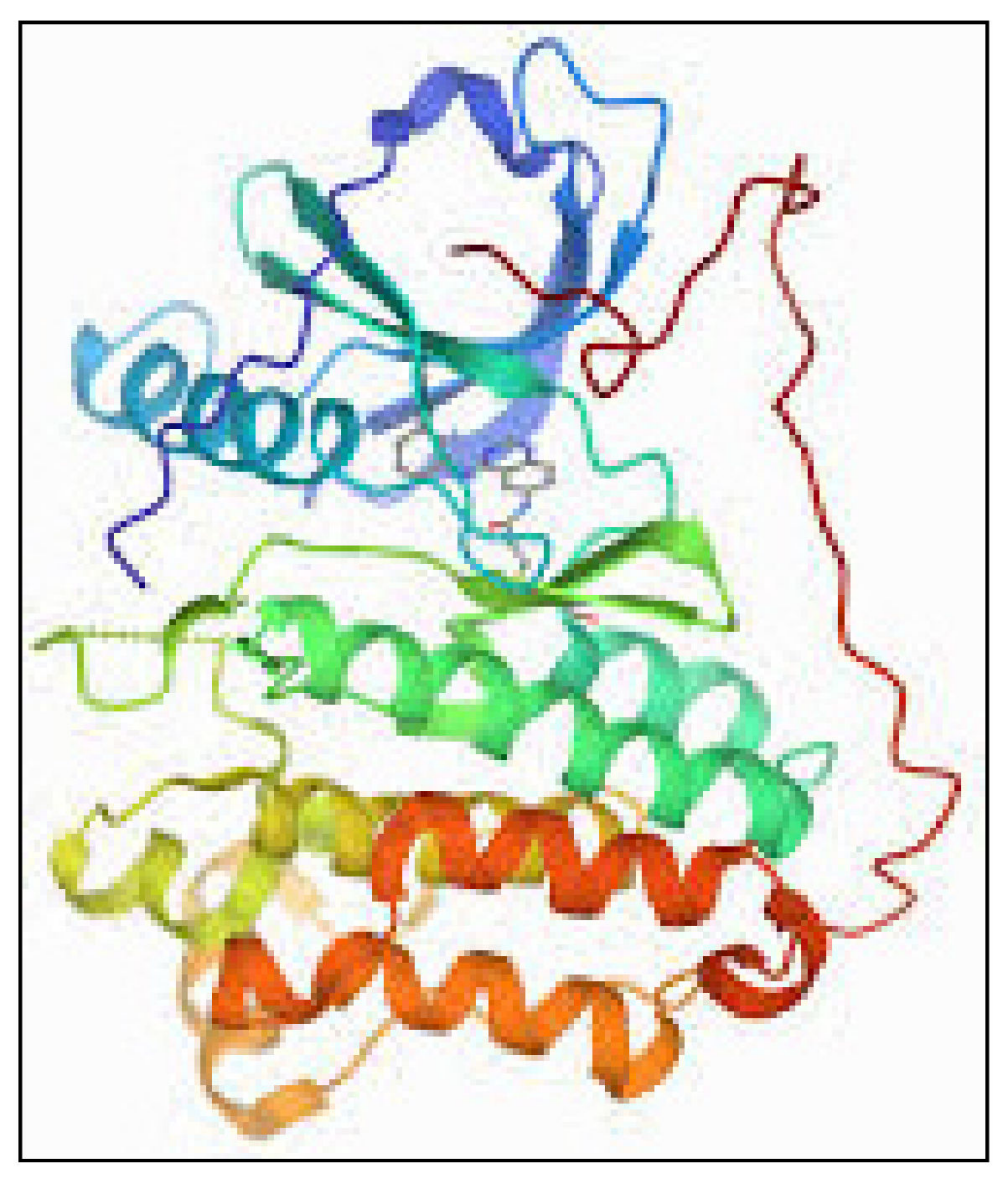
X-ray crystal structure of EGFR tyrosine kinase (PDB: 2J5F) in complex with the 34-JAB.

Towards approaching the in silico studies, the X-ray crystal structure of EGFR tyrosine kinase (PDB: 2J5F) family protein, at a resolution of 3.00 Å, was downloaded (Figure 1) from https://www.rcsb.org/structure/2j5f (accessed on 20 January 2022). The receptor orientations are displayed through the different colored coils (α and β coils) of chain A of the receptor, containing the EGFR kinase receptor domain in complex with an irreversible inhibitor, 34-JAB, which is shown as placed between the blue and the green coiled interaction area as part of the X-ray diffraction-based crystal structure of the tyrosine kinase receptor. It contains 2528 atoms, with 37.67 kDa structure weight where different structural domains of the receptor chain are shown with structural cross-links in different colors (blue, green, red, and yellow) of the structural domains. The protruding side chain (brown color) contains thioether (S-C) linkages with the cysteine residues. The receptor is classified as having global asymmetric–C1 symmetry, and global stoichiometry as monomer–A1 [(2J5F: FirstGlance in Jmol (proteopedia.org, accessed on 15 April 2022), and validation data 2j5f_full_validation.pdf (rcsb.org, accessed on 15 April 2022)].

The EGFR of the tyrosine kinase (TK) family, a transmembrane protein, the active site is defined by the presence of residues Phe^723^, Thr^790^, Lys^745^, Gln^791^, Gly^796^, Leu^792^, Met^793^, Asp^800^, and Asp^855^, which makes it a hydrophobic binding site along with the bound ligand 34-JAB [25].

## 3. Results and Discussion

### 3.1. Chemical Synthesis

**Scheme 1:**
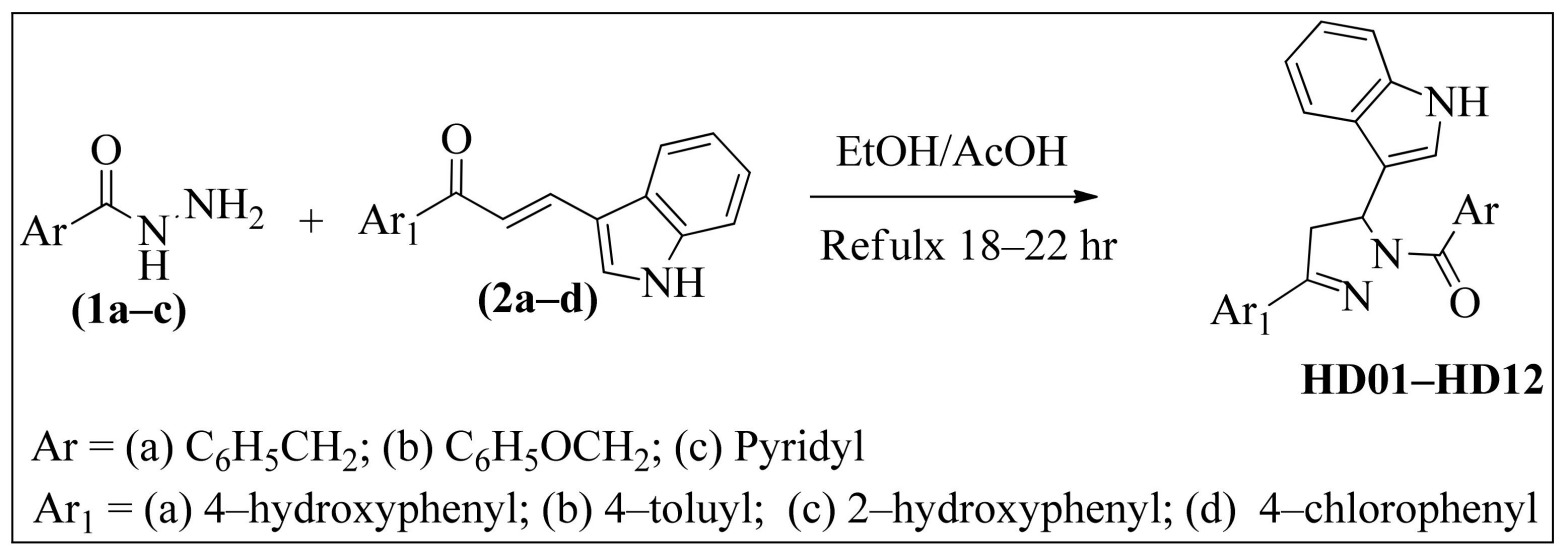
Synthetic scheme for the preparation of pyrazoline derivatives, HD01–HD12.

The synthetic routes used to prepare the starting materials and the title compounds are outlined in Scheme 1. Aromatic acid hydrazide derivatives (**1a**–**c**) were prepared by the reaction between correspondingly substituted aromatic acid, ethanol, and hydrazine hydrate [26]. Indole carboxaldehyde was obtained on condensation with substituted acetophenone in absolute ethanol, and 10% NaOH afforded the corresponding 1-(*substituted*-phenyl)-3-(1*H*-indol-3-yl)-prop-2-en-1-one, chalcanoids **2a**–**d** [27].

The chalcanoids are useful intermediates to generate various compounds with diverse biological activity. The titled indolopyrazolines, HD01-HD12 (Table 1), were synthesized by cyclo-condensation of the chalcanoids **2a**–**d** with hydrazine hydrate derivatives (**1a**–**c**) in absolute ethanol, in the presence of glacial acetic acid, and molecular sieves, where reaction time varied from 18 to 22 h. The products were obtained in varying yields, purified, and characterized.

The highest yield (78.10%) was obtained for the compound HD04. For all the compounds, both the analytical and spectral data analyses were in full agreement with the proposed structures, HD01–HD12. For the ^1^H-NMR spectra of the compounds, the characteristic CH_2_ protons of the pyrazoline ring appeared as a pair of doublets at δ 3.15–3.24 ppm (C_4_-H_A_) and δ 3.68–3.96 ppm (C_4_-H_B_), while the CH proton appeared as a double doublet at δ 5.42, 5.53 ppm (H_X_) due to the vicinal coupling with two magnetically non-equivalent protons of the methylene group at position four (C_4_) of the pyrazoline ring (J_AB_ = 17.51–18.00 Hz, J_AX_ = 4.20–4.80 Hz, J_BX_ = 11.32–12.05 Hz).

All other aromatic and aliphatic protons were observed at expected chemical shift values. The ^13^C–NMR spectra of the compounds exhibited carbonyl carbon signals between the ranges of δ 162.82–165.47 ppm. The chemical shift values of the carbon atoms varied between δ 43.34 and 43.42 ppm for C_4_, δ 60.04–60.24 ppm for C_5_, and δ 151.47–152.38 ppm for C_3_ carbon(s) presence, which corroborated the 2-pyrazoline structural part. The ^1^H-NMR data also supported this. The mass spectral analyses data of the synthesized compounds were found to be in full agreement with the assigned structures. All the compounds showed satisfactory elemental analysis.

### 3.2. Molecular Modelling Studies

The TK protein was prepared by deleting the covalent bond of the bound ligand with the receptor, using a preparation wizard (Maestro, version 8.5, Schrodinger, LLC, New York, NY, USA, 2008). The bond orders were assigned, hydrogen atoms were added, and the water molecules were deleted. The hetero state for the co-crystallized ligand was generated using Epik, protonation state. The optimization of H-bondings of the protein side chains were performed using Protassign^®^. The energy minimizations were carried out using the OPLS-2005 force field. The receptor grid of 12 Å was generated around the active site of EGFR-TK defining the bound ligand by using GLIDE 5.0. The 3D structures of all the ligands were constructed using the Maestro 8.5, and were minimized using the Macromodel minimization panel with the OPLS-2005 force field, and GB/SA water model. LigPrep2.0 module of Schrodinger was used to generate the ionization states at the target pH of 7.0 ± 2.0. The twelve tautomers were generated at this pH, which were employed for ligand dockings. The Xtra precision (XP) mode protocol of GLIDE 5.0 was used for ligand dockings. The molecular docking simulations of the active 1-(5-(1*H*-indol-3-yl)-4,5-dihydro-1*H*-pyrazole-1-yl)-2-phenylethanoid derivatives were performed on the EGFR-TK protein base model (PDB: 2J5F).

A fragment-based development of the primary molecular template was appropriated. The choice of an indole-based chalcanoid infusion with the pyrazole ring as the starting molecular template was envisioned based on several studies on the anti-cancer activities of these two structural templates [28,29,30]. The role of the EGFR on certain chalcanoids has previously been discussed in an earlier report [31]. The modeled compounds, pyrazolinylindoles, at one end, were substituted by benzyl, phenoxy, and pyridyl groups, as the larger moieties selected for the binding at the EGFR-TK site possess the Met^793^ residue. Simultaneously, the other set of substitutions, i.e., chlorophenyl, hydroxyphenyl, and toluyl, were attached nearly at the right angle set of the resultant geometry of the final template in order to interact with the Glu^762^ amino acid residue of the receptor. The attached indole moiety was envisioned to be interacting with the Asp^855^ residue. The binding deep pocket, and active sites containing other residues Phe^723^, Thr^790^, Lys^745^, Gln^791^, Gly^796^, Leu^792^, Met^793^, Asp^800^, and Asp^855^ were also involved in the receptor-ligand interactions with the differently substituted templates. The molecular frameworks of pyrazolinylindoles and the substitutions thereupon helped to deduce the binding pocket disposition, and the geometric specifications of the site.

**Figure 2:**
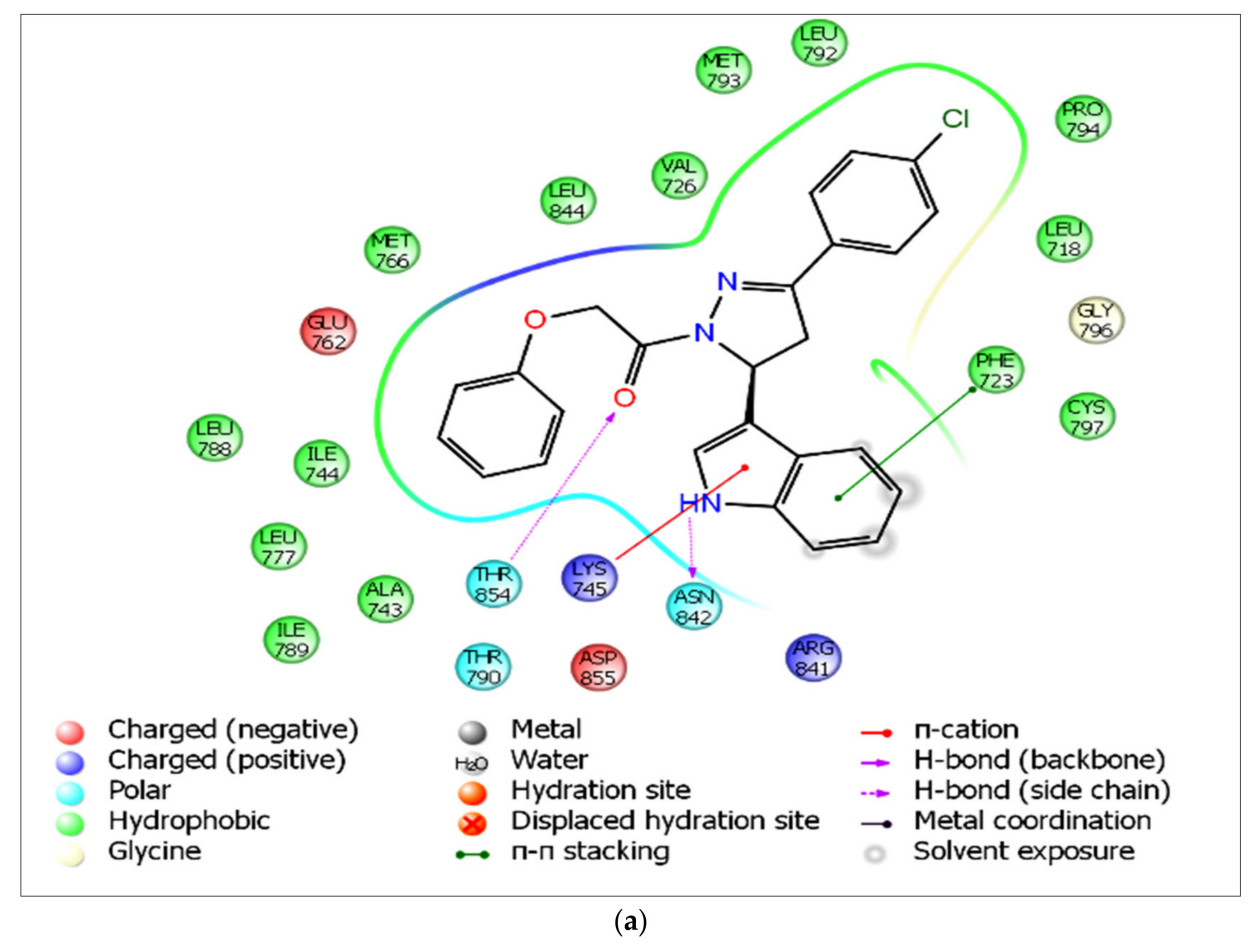

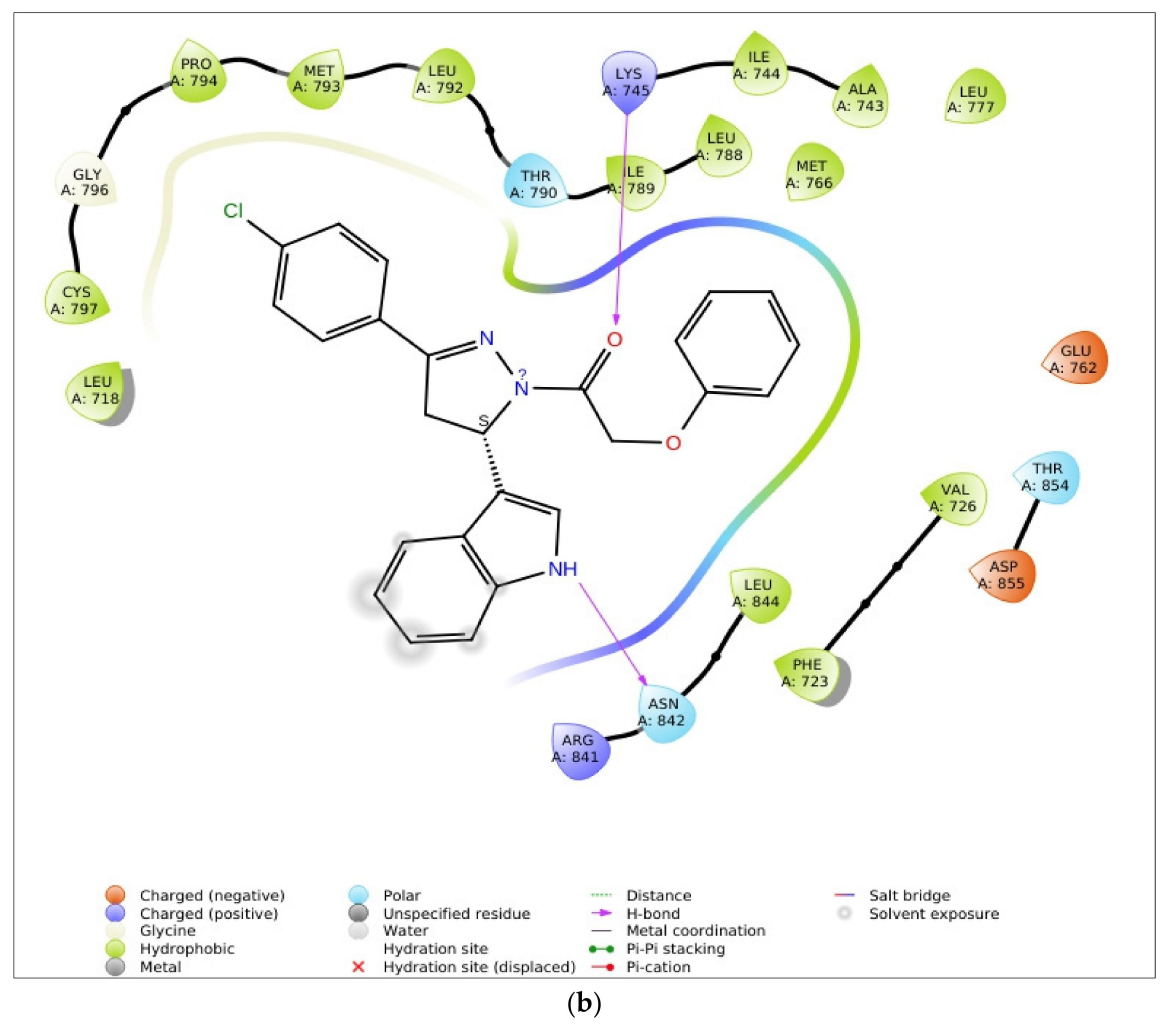
Ligand binding: 2D overlay of the compound HD05 with the active site of EGFR-TK. The binding of HD05 to the active site of the EGFR-TK protein, and the hydrogen binding sites are shown in (**a**,**b**).

**Figure 3:**
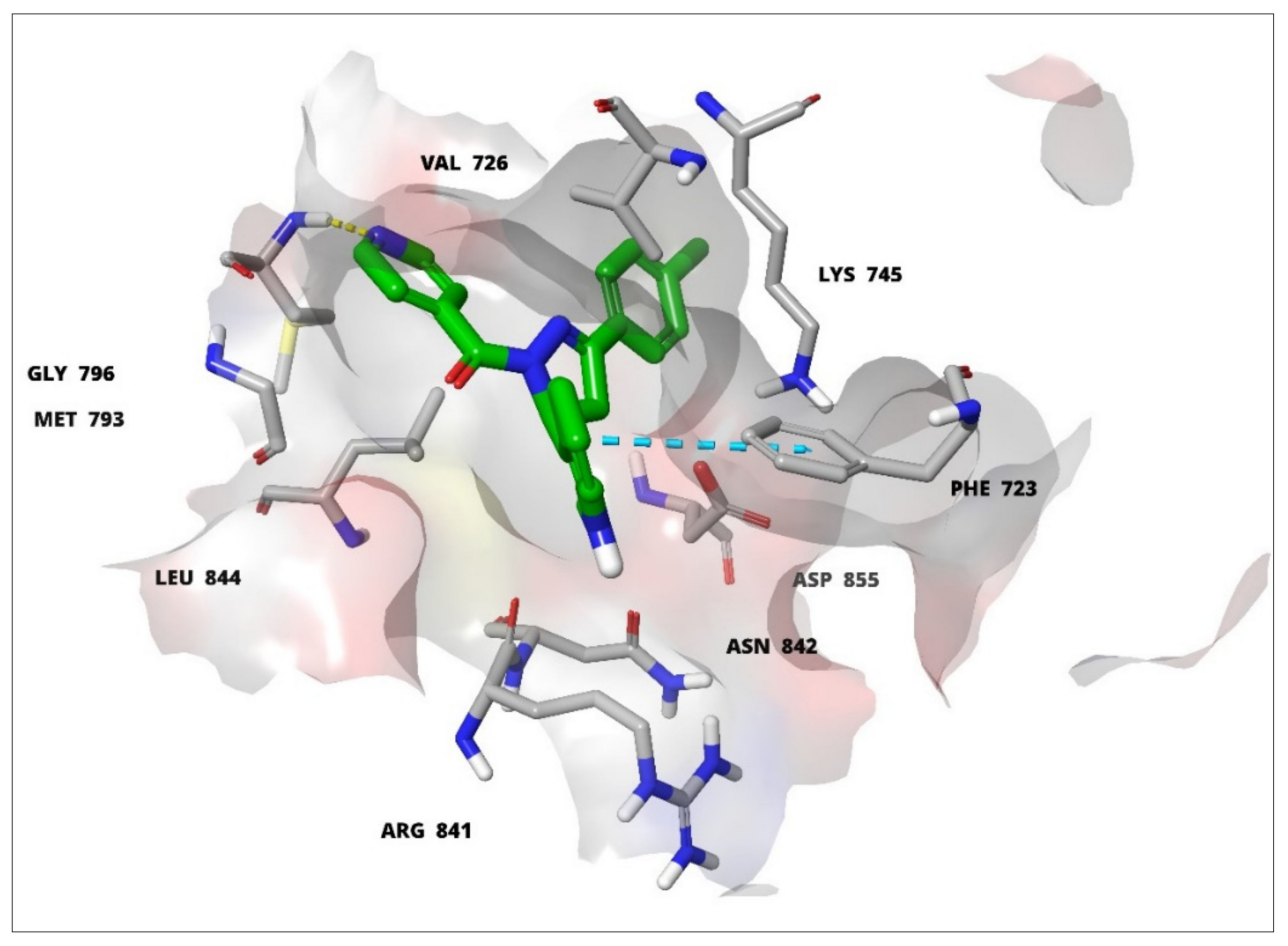
Ligand binding: 3D overlay of the compound HD05 with the active site of EGFR-TK.

The binding of HD05 to the active site of the EGFR-TK protein, and the hydrogen binding sites are shown in Figure 2a,b, while the amino acid bindings, and the interactions as well the structural orientations, together with the 3D binding views of the compound, HD05, are visible in Figure 3. The active compound modeling confirmed the interaction sites, the participating sequence parts, and the geometric orientation of the active molecules. From the IFD (induced-fit docking) studies, the indole of HD05 depicted the π-cation, π–π, and H-bond (hydrogen bonding) interactions with key residues, such as, Arg^841^, Lys^745^, Phe^723^, and Gly^724^ as depicted in Figure 2 and Figure 3, whereas the chlorophenyl group of the compound HD05 structure also shown π-cation interacting with the residue at Lys^745^, halogen-based bond with Thr^790^, and carbamate backbone H-bond interaction with Met^793^ residue. Moreover, the flexibility of Arg^841^, Lys^745^, and Phe^723^ residues were observed in IFD studies, which also favored the bindings with other compounds of the currently synthesized series.

**Figure 4:**
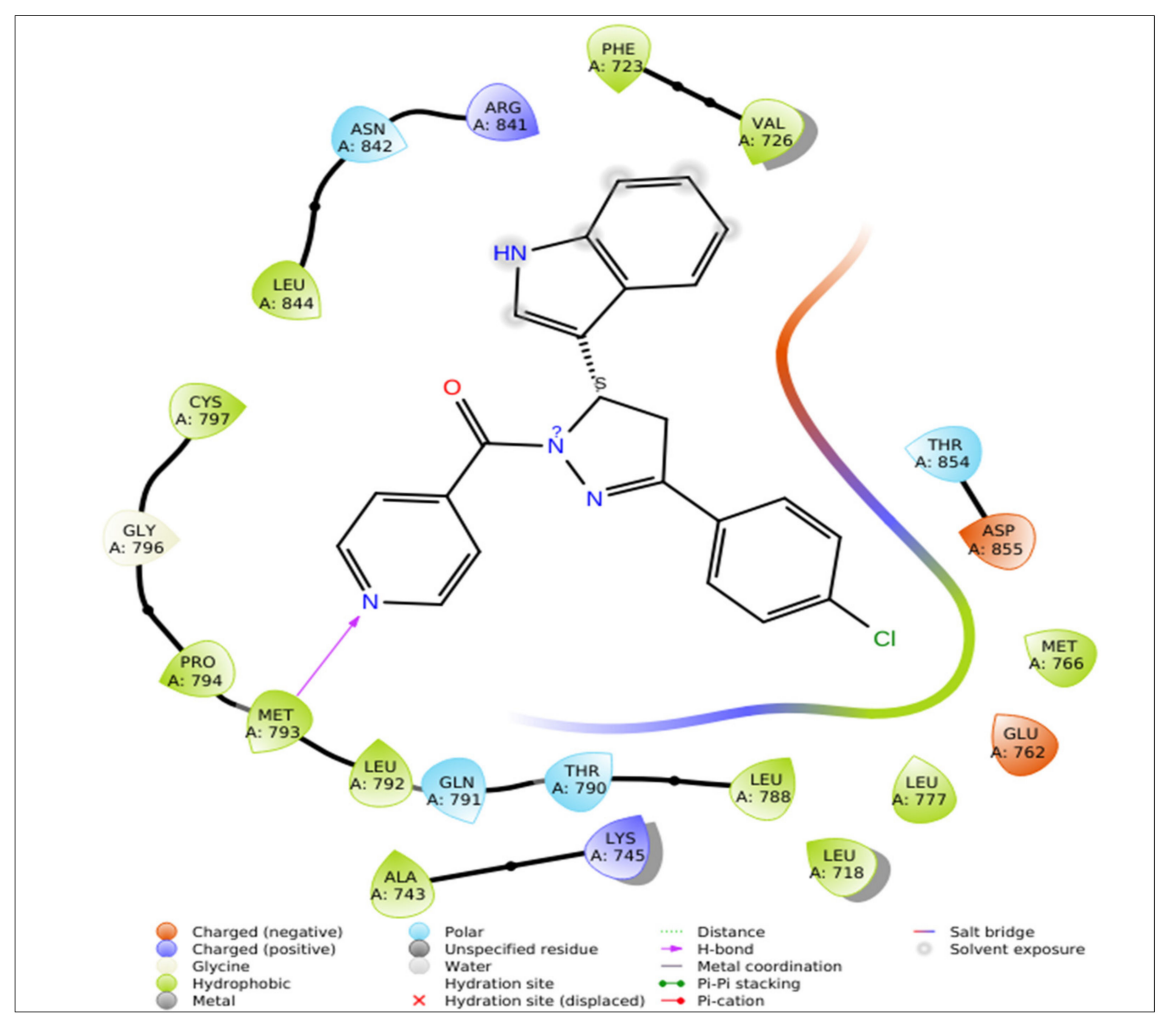
Ligand binding: 2D overlay of the compound HD12 with the active site amino acids of the EGFR-TK.

**Figure 5:**
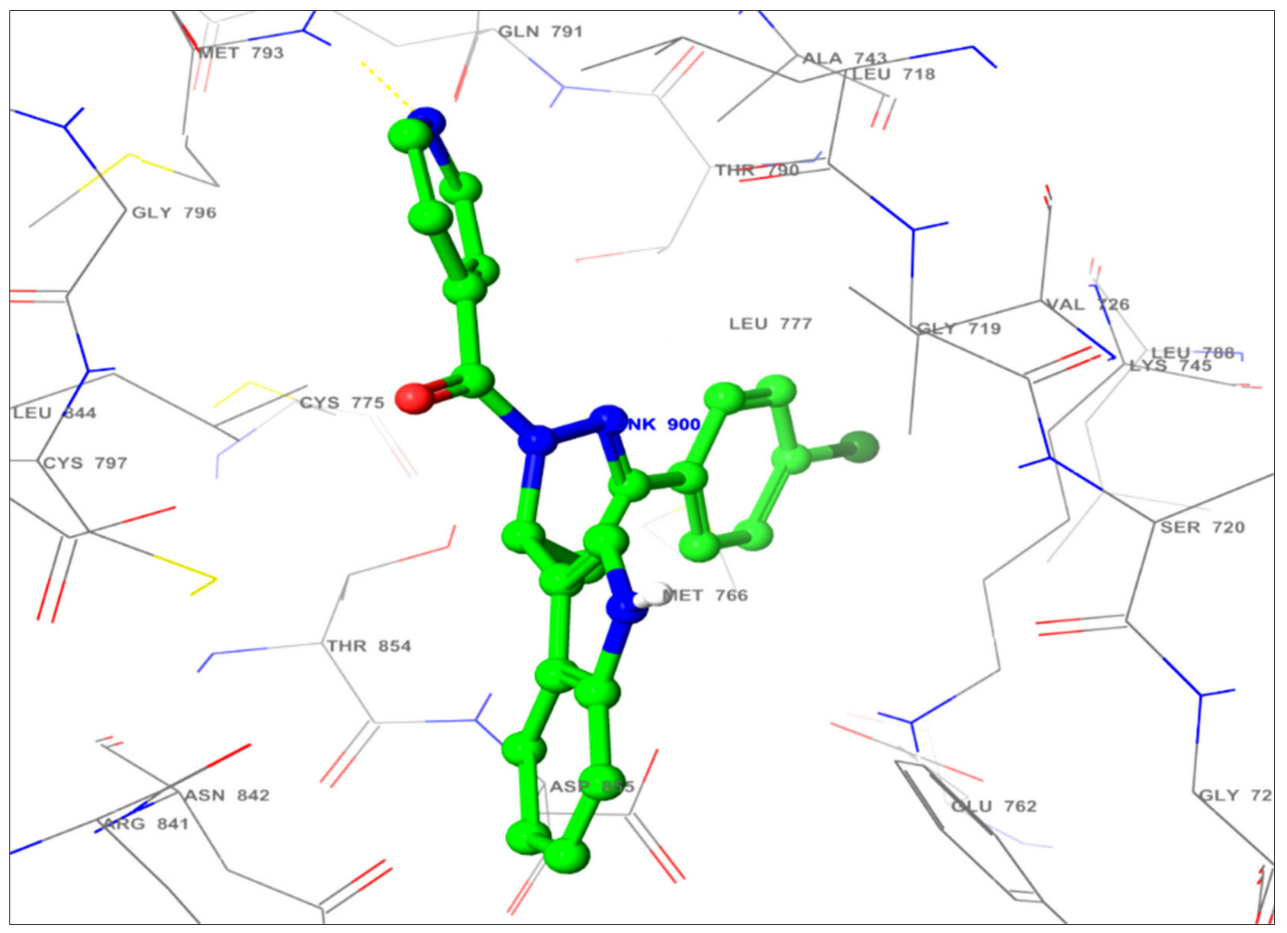
Ligand binding: 3D overlay of the compound HD12 with the interactive binding sites; the yellow dotted lines depict hydrogen bonding. All the active site amino acids residues are shown in solid lines and the green ball and stick model residues indicate the HD12 in EGFR-TK binding. The light blue dotted line indicates the π-π network, and the green dotted line represents the π-cation framework; the representative HD12 compound’s structure is shown in ball and stick model, whereas the interactive residues are depicted in thin sticks with labels, with all other residues shown as normal sticks.

**Figure 6:**
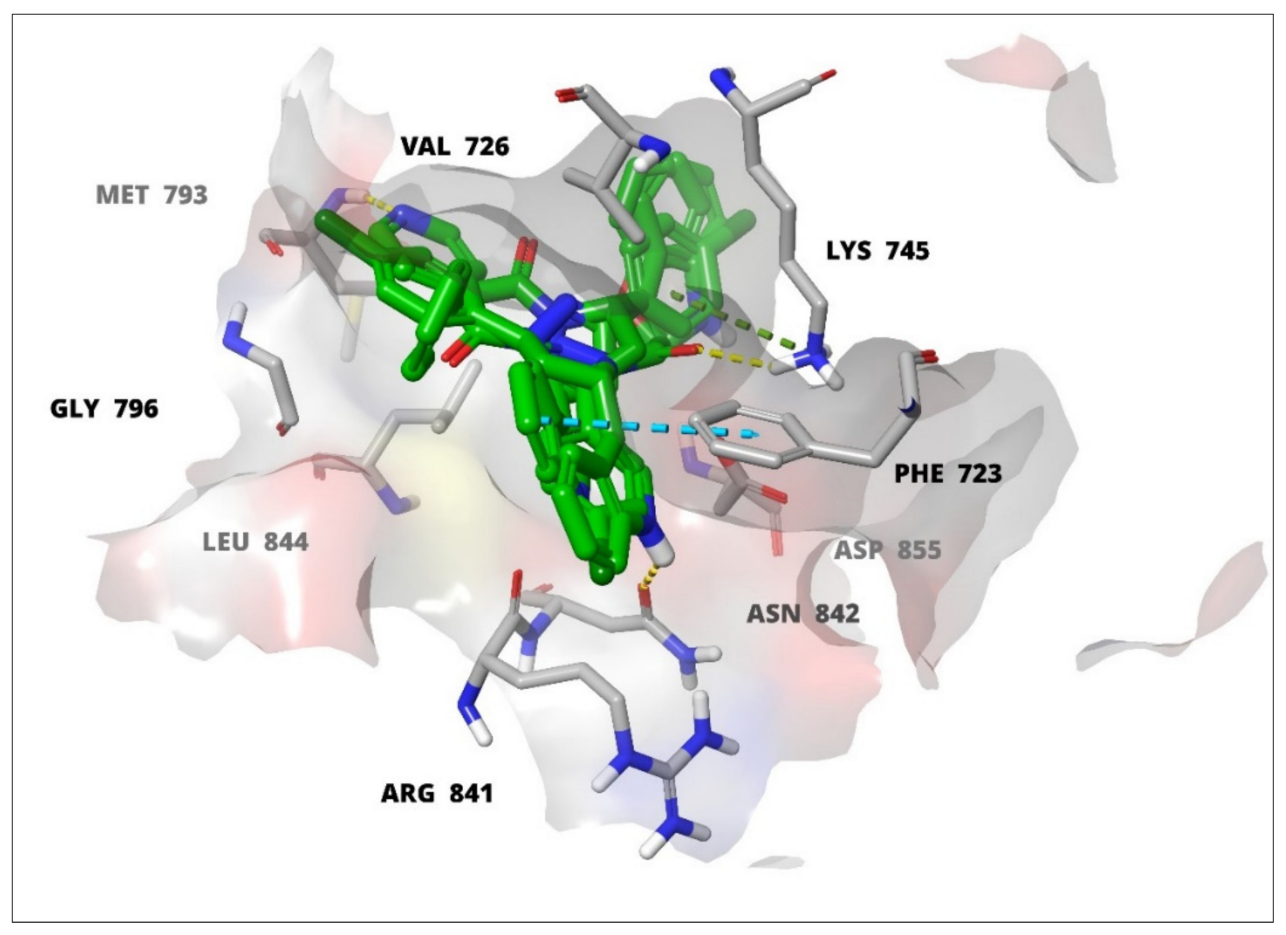
3D in silico view of HD02, HD05, and HD12 at active site of the EGFR-TK.

It was also observed that the pyridine ring of the compounds exhibited backbone hydrogen bonding with crucial residue Met^793^ as shown in Figure 4 and Figure 5, and apart from that, the carbonyl oxygen and nitrogen atoms of the indole nucleus of compounds also showed side-chain hydrogen bonding with Thr^854^ and Asn^842^. An indole nucleus also saw the π-cationic and π–π stacking interactions with residues Lys^745^ and Phe^723^ in the structures, while Figure 6 demonstrated the 3D in silico view at the active site of EGFR tyrosine kinase with the compounds, HD02, HD05, and HD12.

### 3.3. Cell-Line Based Anti-Cancers’ Biological Testings

Preliminary anti-cancers’ screenings were carried out according to the NCI institutional protocols, also described in the Materials and Methods section. The initial testings were performed at single dose levels on leukemia, melanoma, lung, colon, CNS, ovarian, renal, prostate, and breast cancer cell lines. The one-dose data are reported as the mean percent growth inhibition of the treated cancer cells. The reports are in comparison to the control of untreated cells, and are relative at the zero time’s cells’ numbers. The data of anti-cancer screening of the compounds HD02 (NSC: 778247/1), HD05 (NSC: 778249/1), and HD12 (778248/1) are presented in Table 2, which is against nine categories of the cancer types’ cell lines, i.e., leukemia (CCRF-CEM, MOLT4, and SR human lymphoma cancerous cell lines), lungs NSLC (NCI-H460 cell line), melanoma (LOX IMVI cell line), colon (SW620 cell line), CNS (U251 cell line), ovarian (IGROV1 cell line), renal (CAKI-1 and UO31 cell lines), breast (MCF7, MDA-MB231 cell lines), and prostate cancer cell lines (PC-3, DU145).

**Figure 7:**
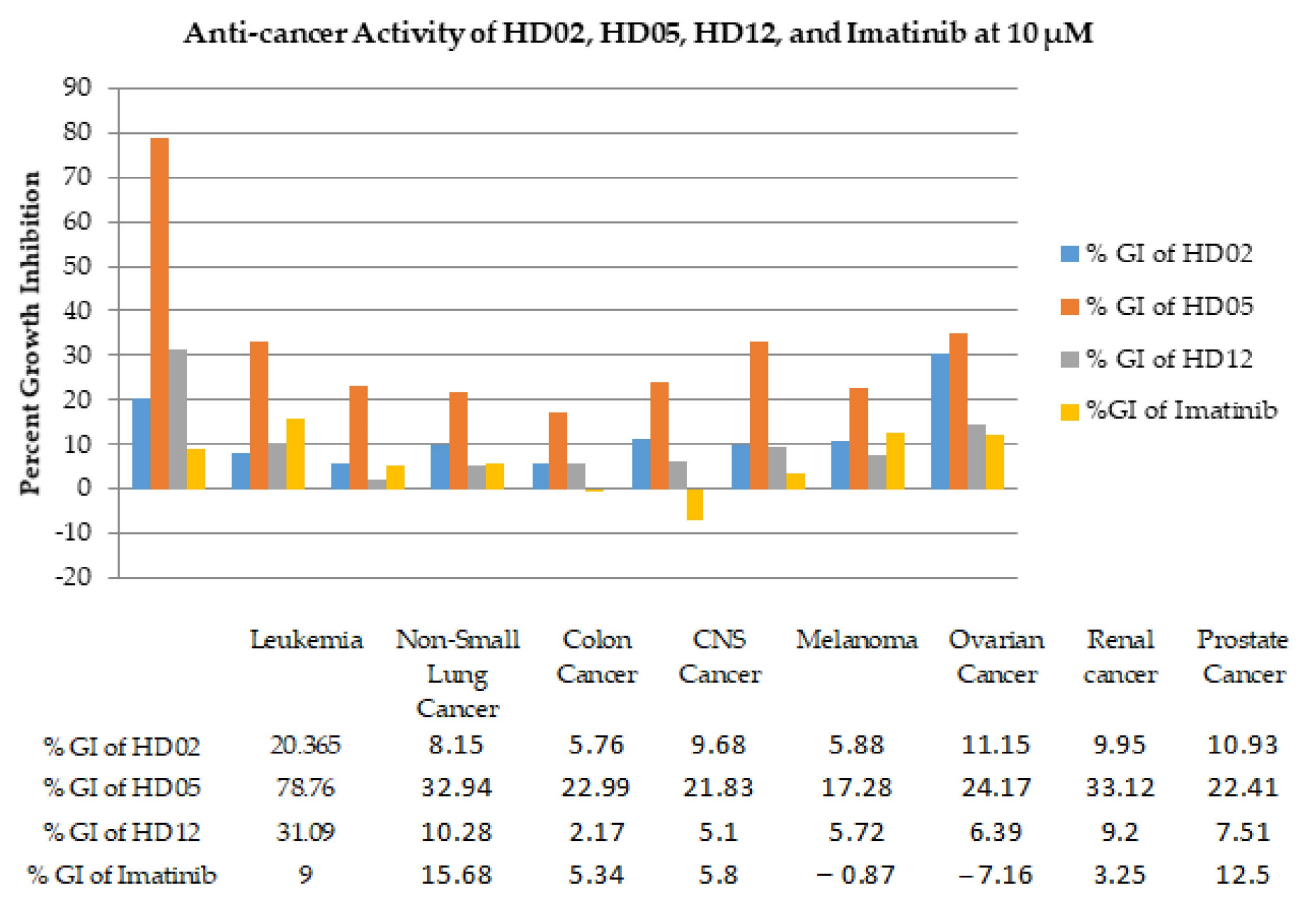
Comparative averaged anti-cancer activity of the compounds HD02, HD05, HD12, and imatinib at doses of 10 µM.

The anti-cancer activity evaluated at NCI, USA, as per the NCI protocol at one dose level assay at 10 µM on nine panels of fifty-six cancer cell lines [24,25,28], are represented in terms of growth percent (GP) and percent growth inhibitions (%GI). The %GI was calculated from GP; that is, %GI = 100-GP. The averaged %GIs of the nine panels of cancer cell lines were calculated in order to compare the anti-cancer activity of compounds HD02, HD05, HD12, and imatinib, as shown in Figure 7. The anti-cancer data of imatinib were retrieved from the NCI website with the drug code NSC 759,854 [32].

Noteworthy cytotoxic effects were recorded for the compound HD05 against all categories of cell lines, i.e., leukemia (MOLT-4, CCRF-CEM), renal (UO-31, CAKI-1, ovarian, breast (MCF7, MDA-MB-231), and non-small lung cancer (NSLC NCI-H460) cell lines (Table 2). Meanwhile, the compound HD02 showed maximum sensitivity against the cancer cell lines, i.e., MCF7 and UO-31 with %GIs of 54.56% (GP = 45.44), and 53.80% (GP = 46.20). The compound HD02 also showed moderate sensitivity against T-47D, and MOLT-4 cell line panels with %GIs at 39.40% (GP = 60.60), and 30.79% (GP = 69.21), respectively. It also exhibited lower sensitivity against MDA-MB-231, SR, MDA-MB-468, and BT-549 cell lines with %GIs at 29.49% (GP = 70.51), 28.96% (GP = 71.04), 26.83% (GP = 73.17), and 25.60% (GP = 74.40)%, respectively. On the rest of the cancer cell lines, it exerted %GIs < 25%. The compound HD02 was also active in two more cell lines, and exhibited anti-cancer activity at over 50% inhibition on renal (UO-31) and breast cancer (MCF7) cell lines.

The compound HD05 registered maximum sensitivity with GI% being > 50%. It was observed on seven cancer cell lines, i.e., MOLT-4, SR, CCFR-CEM, UO-31, IGROV1, MCF7, CAKI-1, and MDA-MB-231, with %GI at 108.31% (GP = −8.31), 89.24% (GP = 10.76), 88.74% (GP = 11.26), 74.30% (GP = 25.70), 69.82% (GP = 30.18), 60.26% (GP = 29.74), 53.97% (GP = 46.03), and 52.27% (GP = 47.83), respectively. The compound HD05 showed moderate sensitivity against T-47D, SW-620, U251, A549, LOX IMVI, ACHN, HOP-92, HCT-15, SN12C, PC-3, NCI-H226, SNB-75, HOP-62, and HCT-116, with %GI at 48.58% (GP = 51.42), 45.52% (GP = 54.48), 41.96% (GP = 58.04), 41.66% (GP = 58.34), 40.90% (GP = 59.10), 39.25% (GP = 60.75), 37.87% (GP = 62.13), 37.87% (GP = 62.13), 37.31% (GP = 62.69), 36.78% (GP = 63.22), 34.82% (GP = 65.18), 33.44% (GP = 66.52), 33.42% (GP = 66.58), and 31.50% (GP = 68.50), respectively. The compound HD05 also exhibited lower sensitivity against OVCAR-8, UACC-257, 786-O, K-562, BT-549, SF-268, A498, NCI-H522, KM12, and MALME-3M cell lines, with %GIs observed at 29.57% (GP = 70.43), 29.10% (GP = 70.90), 28.98% (GP = 71.02), 28.75% (GP = 71.25), 28.66% (GP = 71.34), 28.09% (GP = 71.91), 27.93% (GP = 72.07), 26.08% (GP = 73.92), 25.95% (GP = 74.05), and 25.90% (GP = 74.10), respectively. On rest of the cancer cell lines, it exerted %GIs < 25%. The compound HD05 also exerted lethal effects on MOLT-4 cancer cell lines with %GI of 108.31 (GP = −8.31)%. The anti-cancer activity of the compound HD05 was found to be promising (Table 2 and Figure 7). The compound HD05 showed maximum anti-cancer activity on all panels of cancer cell lines as compared to the compounds, HD02, HD12, and imatinib referral standard (Figure 7).

The compound HD12 showed moderate sensitivity against SR and CCRF-CEM cell lines, with %GIs of 44.52% (GP = 57.48), and 43.29% (GP = 56.71), respectively. The compound HD12 also showed lower sensitivity against MCF7, MDA-MB-231, and T-47D cell lines, with lower %GIs of 29.85% (GP = 70.15), 28.65% (GP = 70.35), and 25.20% (GP = 74.80)%, respectively. On the rest of the cancer cell lines, it exerted %GIs < 25%. Nonetheless, the compound HD12 was found to be active at 54.83% of GI on renal (UO-31) cancer cell line.

Thus, the compounds HD02, HD05, and HD12 hold promise for the future and can serve as a template, and new molecular leads for further development. The compound HD05 showed higher anti-cancer activity than imatinib on all nine cell lines panels, leukemia, melanoma, ovarian cancer, CNS, renal cancer, lung cancer, colon cancer, and breast cancer.

## 4. Conclusions

Compounds HD02, HD05, and HD12, showed higher cell line growth inhibitions against nine panels of cell line categories constituting fifty-six cell lines. The compound HD05 was mostly active against all the cancer cell line types, and showed better activity against the leukemia cell line, MOLT4. This compound can serve as a template for new molecular leads toward further development.

## Figures and Tables

**Table 1:** Synthesized compounds, HD01–HD12.

Compound	Substitution Ar ^1^	Substitution Ar
HD01	4-Hydroxyphenyl	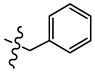
HD02 *	4-Toluyl	
HD03	2-Hydroxyphenyl	
HD04	4-Chlorophenyl	
HD05 *	4-Chlorophenyl	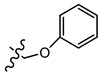
HD06	4-Toluyl	
HD07	2-Hydroxyphenyl	
HD08	4-Hydroxyphenyl	
HD09	4-Hydroxyphenyl	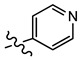
HD10	4-Toluyl	
HD11	2-Hydroxyphenyl	
HD12 *	4-Chlorophenyl	

* Preliminary biological testings found higher levels of anti-cancer activity in these compounds; Ar ^1^: aromatic substituents on 5th carbon as shown in synthetic Scheme 1.

**Table 2:** Anti-cancer activity of compounds, HD02, HD05, and HD12 against 56 NCI cancer cell lines.

Structures		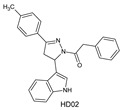		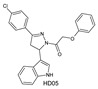		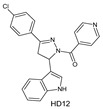	
Panels	Cell Lines	HD02		HD05		HD12	
		GP ^+^	%GI ^++^	GP	%GI	GP	%GI
Leukemia	CCRF-CEM	84.23	15.77	11.26	88.74	56.71	43.29
	K-562	94.06	5.94	71.25	28.75	88.34	11.66
	MOLT-4	69.21	30.79	−8.31	108.31	75.08	24.92
	SR	71.04	28.96	10.76	89.24	57.48	44.52
Non-Small Lung Cancer	A549/ATCC	91.82	8.18	58.36	41.66	95.40	4.60
	HOP-62	78.86	21.14	66.58	33.42	75.10	24.90
	HOP-92	125.23	−25.23	62.13	37.87	94.69	5.31
	NCI-H226	85.76	14.24	65.18	34.82	82.72	17.28
	NCI-H23	82.32	17.68	76.06	23.96	85.92	14.08
	NCI-H322M	87.09	12.91	86.43	13.57	96.29	3.71
	NCI-H460	97.31	2.69	47.85	52.15	96.81	3.19
	NCI-H522	76.40	13.60	73.92	26.08	80.83	9.17
Colon Cancer	Colo205	100.95	−0.95	86.89	13.11	97.86	2.14
	HCC-2998	105.08	−5.08	102.59	−2.59	105.27	−5.27
	HCT-116	87.46	12.54	68.50	31.50	94.28	5.72
	HCT-15	80.91	19.09	62.13	37.87	93.56	6.44
	HT29	93.44	6.56	90.38	9.62	101.08	−1.08
	KM12	91.57	8.43	74.05	25.95	96.75	3.25
	SW-620	100.21	−0.21	54.48	45.52	96.01	3.99
CNS Cancer	SF-268	87.31	12.69	71.91	28.09	94.19	5.81
	SF-295	88.67	11.33	92.08	7.92	96.97	3.03
	SF-539	87.24	12.76	87.08	12.92	97.05	2.95
	SNB-19	104.05	−4.05	93.31	6.69	105.40	−5.40
	SNB-75	87.00	13.00	66.56	33.44	82.41	17.59
	U251	87.64	12.36	58.04	41.96	93.37	6.63
Melanoma	LOX IMVI	83.15	16.85	59.10	40.90	86.90	13.10
	MALME-3M	86.75	13.25	74.10	25.90	96.65	3.35
	M14	91.89	8.11	83.11	16.89	95.25	4.75
	MDA-MB-435	106.49	−6.49	93.82	6.18	99.89	0.11
	SK-MEL-2	87.80	12.20	85.33	14.67	82.96	17.04
	SK-MEL-28	112.91	−12.91	103.52	-3.52	107.05	−7.05
	SK-MEL-5	93.37	6.63	85.51	14.49	93.26	6.74
	UACC-257	99.55	0.45	89.06	10.94	95.95	4.05
	UACC-62	85.17	14.83	70.90	29.10	90.53	9.47
Ovarian Cancer	IGROV1	75.59	24.41	30.18	69.82	79.27	20.83
	OVCAR-3	92.32	7.68	77.86	22.14	95.48	4.52
	OVCAR-5	91.79	8.21	112.11	−12.11	105.36	−5.36
	OVCAR-8	93.68	6.32	70.43	29.57	98.46	1.54
	NCI/ADR-RES	92.96	7.04	83.00	17.00	96.98	3.02
	SK-OV-3	84.72	13.28	81.39	18.61	84.19	13.81
Renal cancer	786-0	103.27	−3.27	71.02	28.98	104.02	−4.02
	A498	75.77	24.23	72.07	27.93	80.54	19.46
	ACHN	89.39	10.61	60.75	39.25	94.83	5.17
	CAKI-1	93.03	6.97	46.03	53.97	90.21	9.79
	RXF 393	108.82	−8.82	103.23	−3.23	111.61	−11.61
	SN12C	93.05	6.95	62.69	37.31	92.95	7.05
	TK-10	110.87	−10.87	93.59	6.41	107.04	−7.04
	UO-31	46.20	53.80	25.70	74.30	45.17	54.83
Prostate Cancer	PC-3	76.57	23.43	63.22	36.78	82.77	17.23
	DU-145	101.58	−1.58	91.97	8.03	102.22	−2.22
Breast cancer	MCF7	45.44	54.56	39.74	60.26	70.15	29.85
	MDA-MB-231/ATCC	70.51	29.49	47.73	52.27	71.35	28.65
	HS 578T	93.97	6.03	93.73	6.27	100.69	−0.69
	BT-549	74.40	25.60	71.34	28.66	92.75	7.25
	T-47D	60.60	39.40	51.42	48.58	74.80	25.20
	MDA-MB-468	73.17	26.83	85.72	14.28	102.09	−2.90
Mean		88.21	11.89	69.80	30.20	90.55	9.45

GP ^+^ = Growth percent; %GI ^++^ = Growth inhibition.

## Data Availability

Data is contained within the article.
